# Progress Towards Elimination of Trachoma in Kenya 2017–2020

**DOI:** 10.1080/09286586.2023.2280987

**Published:** 2024-02-06

**Authors:** D Ilako, S Mwatha, Barasa E Wanyama, M Gichangi, J Bore, R Butcher, A Bakhtiari, S Boyd, R Willis, AW Solomon, T Watitu, D Chelanga, P Nyakundi, EM Harding-Esch, SH Matendechero

**Affiliations:** a Department of Ophthalmology, University of Nairobi, Nairobi, Kenya; b Neglected Tropical Diseases Unit, Ministry of Health, Nairobi, Kenya; c Ophthalmic Services Unit, Ministry of Health, Nairobi, Kenya; d Kenya National Bureau of Statics, Nairobi, Kenya; e Clinical Research Department, London School of Hygiene & Tropical Medicine, Keppel Street, London, UK; f International Trachoma Initiative, Task Force for Global Health, Atlanta, Georgia, USA; g Global Neglected Tropical Diseases Programme, World Health Organization, Avenue Appia 20, Geneva, Switzerland

**Keywords:** Blindness, elimination, Kenya, neglected tropical disease, prevalence, Trachoma

## Abstract

**Purpose:**

Trachoma is endemic in Kenya. Since baseline trachoma surveys in 2004, a concerted programme has been undertaken to reduce the prevalence of disease. Here, we report on trachoma prevalence surveys carried out between 2017 and 2020 after interventions were implemented in some areas for trachoma elimination purposes.

**Methods:**

A total of 48 cross-sectional population-based trachoma prevalence surveys were conducted in 39 evaluation units (EUs; covering 45 subcounties) of Kenya between 2017 and 2020. Thirty EUs were surveyed once and nine EUs were surveyed twice over this period. Individuals ≥ 1 year old were assessed for trachomatous inflammation—follicular (TF), trachomatous inflammation—intense (TI) and trichiasis. Data were collected on household access to water, sanitation and hygiene (WASH).

**Results:**

A total of 147,573 people were examined. At the end of 2020, in the 39 EUs surveyed, the prevalence of TF in 1–9-year-olds was ≥5% in 11 EUs and the prevalence of trichiasis unknown to the health system in individuals aged ≥15 years was ≥0.2% in 25 EUs. A small minority of households (median <50% for all indicators) had access to improved WASH facilities.

**Conclusion:**

Kenya has made excellent progress towards elimination of trachoma as a public health problem. However, there is more work to do. Between one and three rounds of antibiotic mass drug administration are required in 11 EUs. Sustained investment in surgical provision, continued TT case-finding, promotion of facial cleanliness and environmental improvement are required throughout the surveyed area.

## Introduction

Trachoma involves a chronic conjunctivitis caused by ocular infection with *Chlamydia trachomatis*.^1^ Trachoma is the leading infectious cause of blindness worldwide,^2^ and is targeted for global elimination as a public health problem by 2030.^3^ The World Health Organization (WHO) recommends the use of the SAFE (surgery, antibiotics delivered as mass drug administration [MDA], facial cleanliness, environmental improvement) strategy to reduce the burden of trachoma.^4,5^ Elimination as a public health problem is achieved when: (1) the prevalence of trachomatous inflammation—follicular (TF) in children aged 1–9 years is <5% in each formerly endemic district; (2) the prevalence of trachomatous trichiasis (TT) unknown to the health system in those aged ≥15 years is <0.2% in each formerly endemic district, and (3) there is a system in place to manage incident cases of TT.^6^

Trachoma has been a major contributor to blindness in Kenya in the past.^7,8^ Considerable effort has been made to reduce the prevalence of disease. The SAFE strategy was piloted in Kajiado county, where in 2004–05, TF and TT were found to be lower in prevalence following provision of MDA and surgical services to a focused area around Shompole.^9^ Large, pre-intervention (trachoma baseline, TBL) surveys were carried out in suspected-endemic areas nationwide using WHO-recommended methodologies between 2004 and 2012.^10,11^ Since this TBL mapping was carried out, the SAFE strategy has been implemented throughout endemic areas. Where MDA has been implemented, coverage has reportedly been >80%. In collaboration with the water, sanitation and hygiene (WASH) sector, the trachoma programme has also been mobilizing resources towards increasing access to potable water, improved sanitation and behaviour change communication. From 2010 to 2011, TT cases were managed using TT outreach camps which relied on individuals coming forward for treatment at screening sites. As cases of TT dwindled, however, it became more cost-effective to train case-finders to identify TT cases at household level and link them to surgeons for confirmation and management. Case-finding was implemented between 2016 and 2019, after which full geographic coverage (FGC) approaches were adopted as proof that every household member had a chance to be examined and linked to eye health teams where required for confirmation and management of TT. Research studies in high-risk populations suggest some positive outcomes, such as increased face washing.^9,12–14^ Challenges for the programme have been identified: first, more TT surgeries were required to reduce the TT prevalence to <0.2% among adults 15+ than originally estimated; second, cross-border migration from neighbouring countries was highlighted as a potential source of re-infection after treatment; and third, water availability was poor in many mapped areas.

Here, data are presented from subsequent TBL and trachoma impact surveys (TIS) carried out in Kenya in 2017–2020, to assess prevalence of trachoma in areas across Kenya which were either known, or suspected to be, trachoma-endemic. The aim of this work was to determine whether future trachoma elimination interventions are required in the surveyed areas.

## Methods

### Study ethics and participant consent

These surveys were approved by the Kenya Medical Research Institute and the Kenya National Review Ethics Committee. Tropical Data ethical approval to support these surveys was provided by the London School of Hygiene & Tropical Medicine Ethics Committee (reference: 16105). Participants were provided information in the local language both verbally and in writing before being invited to participate. Participants (or their parent/guardian, if <18 years) consented verbally. Children aged 13–17 years provided assent. Each team contained a village guide who was familiar with the local area to ensure local cultures were respected. Village and house-hold heads were asked for permission to visit before approaching each village/household.

### Study design and participant selection

Population-based prevalence surveys for trachoma were conducted using consecutive versions of a standardised methodology.^15–17^ Surveys in 2017 − 2019 utilised the 2017 Tropical Data methodology (version 2)^17^ and surveys in 2020 utilised the 2019 Tropical Data methodology (version 3).^16^ These two versions had key differences in the definition of TT and the recording of handwash stations (outlined below). Results are reported from TBL surveys (where no district-level prevalence data had previously been collected), new TBL surveys (NBL; where prevalence surveys had previously been conducted, but no interventions had been carried out in recent years and the contemporary prevalence of disease was unknown), and trachoma impact surveys (TIS) (where interventions had been recently carried out as recommended).

Areas for surveying were divided into evaluation units (EUs), defined by WHO for trachoma elimination purposes as the normal administrative unit for healthcare management, with a population between 100,000–250,000 people.^5^ In Kenya, this was mostly equivalent to a one subcounty, but occasionally equivalent to more than one subcounty. For the TBL surveys presented here, the target sample size was calculated to estimate a TF prevalence of 10% (±3%) among children aged 1–9 years in each EU. Assuming a design effect of 2.65, 1,222 children were required in each TBL and NBL EU.^18^ For TIS, the target sample size was calculated to estimate a TF prevalence of 4% (±2%) among children aged 1–9 years in each EU. Assuming a design effect of 2.63, 1,164 children were required in each EU.^15^ Both calculations include an inflation factor to accommodate a 20% non-response rate.

Individuals were selected for inclusion using two-stage cluster sampling. The primary sampling unit (PSU) was the village, selected systematically using a probability-proportional-to-size methodology from a list of all the villages in the EU. The secondary sampling unit was the household, selected using compact segment sampling. The number of secondary sampling units was fixed at 30, the number which could be feasibly visited by a field team in one day. All household residents aged ≥1 year were eligible for inclusion. Simulated sampling of other datasets suggested acceptable precision would be reached using a maximum of 30 PSUs,^19^ therefore 30 PSUs were targeted in these surveys.

### Clinical examination

All eligible and consenting survey participants were examined for signs of trachoma using 2.5× binocular loupes. Follicle size guides were also used in 2020 to support TF diagnosis.^20^ Graders had attended taught seminars on trachoma and reviewed photographs of cases with a Tropical Data-certified grader trainer, and were required to achieve a kappa score of ≥0.7 when grading photographs and 50 live subjects, of whom at least 5 had TF. Each grader also received one supervisory visit in the field.

Surveys in 2017 − 2019 used the original version of the WHO simplified trachoma grading system,^21^ and surveys in 2020 used the amended version of the WHO simplified trachoma grading system.^22^ One key difference in the grading systems is in the grading of TT: in 2017 − 2019 surveys, TT was called when deviated eyelashes originated from the upper and/or lower eyelid.^23^ In 2020 surveys, only upper-eyelid trichiasis was considered to be TT. In all surveys, individuals with trichiasis were asked questions about previous management of their trichiasis. The presence or absence of trachomatous scarring (TS) was also recorded for individuals with trichiasis. Individuals found to have active trachoma (TF and/or TI) were treated free-of-charge with 1% tetracy-cline eye ointment and adults with TT were referred for appropriate management.

### Household information

Data recorders in each team underwent training to accurately record the results of the clinical examination and household WASH access, including being able to identify different types of latrine and water source. A version of the WHO/United Nations Children’s Fund Joint Monitoring Program (JMP) Core Questions for Households adapted for trachoma surveys was used.^18,24^

During the survey, the questionnaire was delivered to senior members of each household, and water sources and latrines were categorised by data recorders, where possible by direct observation. WASH data were classified as improved or unimproved according to the JMP definitions. Surveys in 2017−2019 only recorded whether households had a handwash station within 15 m of a latrine; where there was no latrine, no handwash data were collected. Surveys in 2020 recorded the presence or absence of a handwash station in or near the dwelling for all participating households.

### Data recording, storage and analysis

Data were recorded using an Android smartphone application based on Open Data Kit software. Completed data collection forms were checked and cleaned by Tropical Data (tropicaldata.org) data managers throughout the data collection period. TT cases were classed as ‘unknown to the health system’ if they reported not having been offered management (surgery and/or epilation) in at least one eye, or if they couldn’t remember being offered management.^25^ The prevalence of TF was adjusted for age in one-year age brackets using the most recent census. The prevalence of TT unknown to the health system was adjusted for age and gender in five-year bands.^26^ Prevalence estimates were generated and adjusted using the Global Trachoma Mapping project (GTMP) approach.^18^

The association between TF, household- and individual-level risk factors was determined using a multi-level mixed-effects logistic regression model.^27^ EU and primary sampling unit of residence were included as random-effect variables in the final multivariable model. Variables were assessed for association with TF in univariable analysis and for collinearity with other variables prior to inclusion in the final multivariable model. Washing water source and drinking water source variables were identified to be collinear (Pearson correlation coefficient > 0.8) therefore only washing water source was used in the analyses. The contribution of each variable was assessed by likelihood ratio testing between models with and without the variable in question. Before conducting regression analyses, children living in households where the facilities were classified as ‘other’ were removed from the analysis (*n* = 1,724) as it was not possible to assign them to a particular category.

The association between gender and being unknown to the health system was assessed with a chi-squared test in a separate analysis.

## Results

### Survey demographics

A total of 147,573 individuals aged ≥1 year were examined for trachoma in 48 surveys, representing 95% of the enumerated population (155,634 people). Of the people who were enumerated but did not take part, the majority (7,803/8,061) were absent at the time of the teams’ visits. 244/8,061 (3%) refused and 14/8,061 (0.2%) recorded another reason for not taking part (Supplementary Table S1). The surveys reported in this manuscript included one TBL, 11 NBLs, 27 first TIS (TIS1) and nine second TIS (TIS2). TIS2 is a second round of impact surveys required after the first programmed period of annual MDA was not associated with the subsequent TIS demonstrating a TF prevalence <5%.

### Clinical examination findings

**Figure 1:**
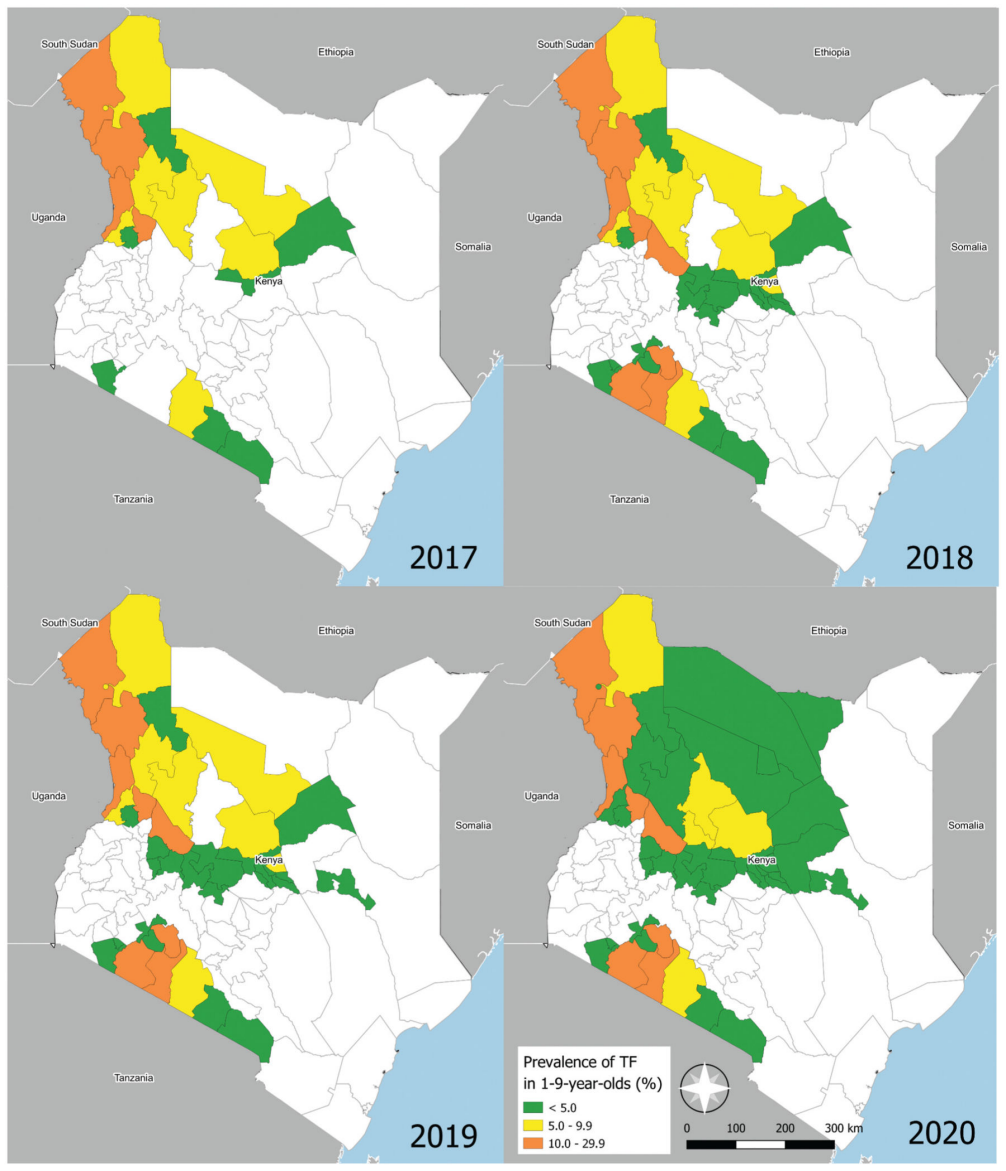
Adjusted prevalence of trachomatous inflammation—follicular (TF) in Kenya. Counties coloured in white were not surveyed. The boundaries and names indicated and the designations used on this map do not imply the expression of any opinion of any kind on the part of the authors, or the institutions with which they are affiliated, concerning the legal status of a country, territory, city or region or its authorities, or concerning the delimitation of its borders.

**Figure 2:**
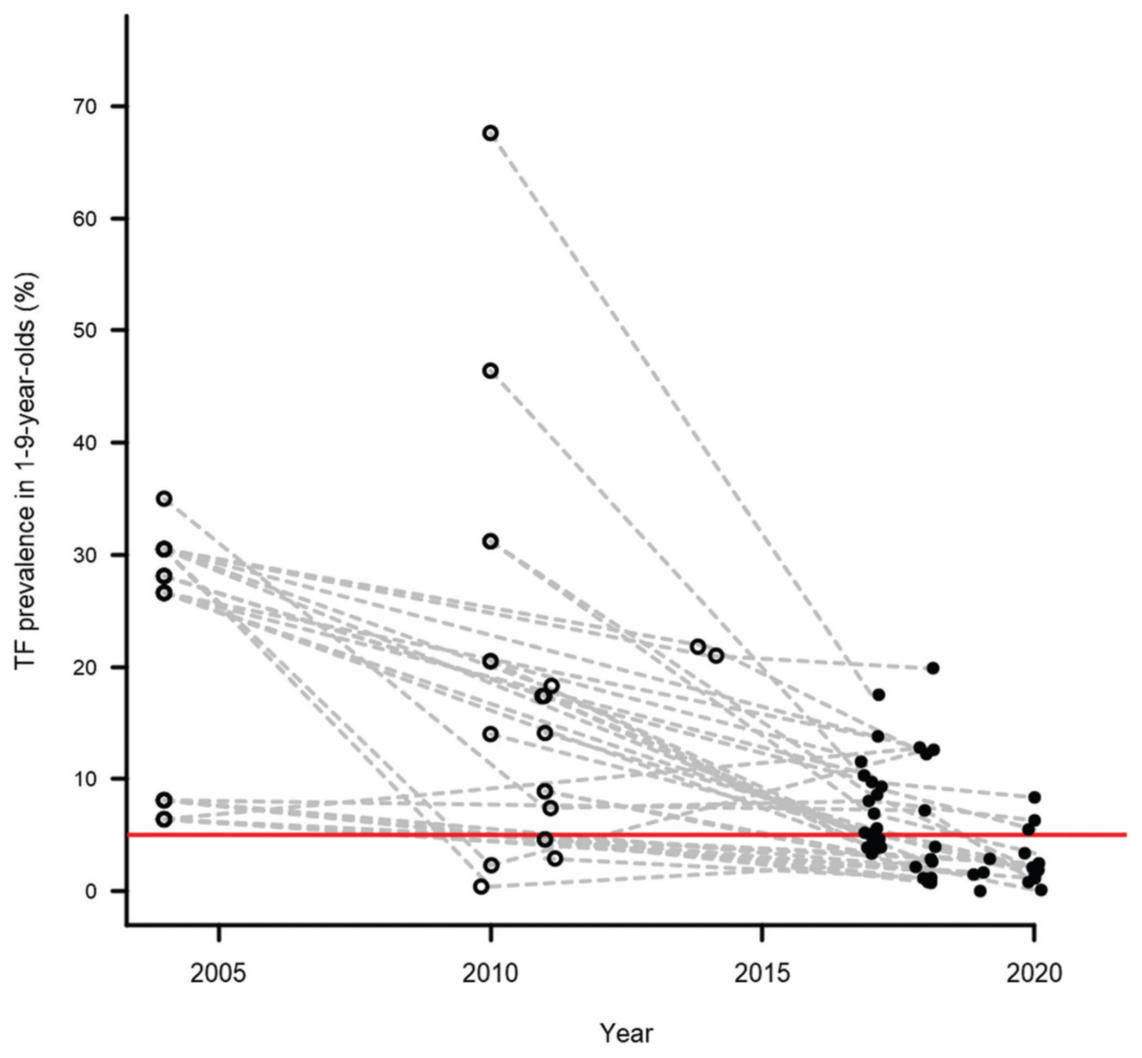
Prevalence of trachomatous inflammation—follicular (TF) in children aged 1–9 years in Kenya. Prevalence data from 2004 are published elsewhere.^10^ data from 2010–2014 were taken from unpublished reports of programmatic activities. The horizontal red line indicates the 5% TF threshold. To ensure the prevalence estimates are clearly visible, the precision of each estimate is not illustrated on this plot.

There were 70,143 children aged 1–9 years examined in 48 surveys. In those children, 4,100 cases of TF and 711 cases of TI were identified. The prevalence of TF ranged from 0% in Mbalambala/Garissa Township of Garissa county to 19.9% in Segment 5 of Narok county (Supplementary Table S2). There were 11 TBL and NBL surveys in which the TF prevalence was <5.0%. One NBL survey returned a TF prevalence ≥10.0%. There were 11, nine and seven TIS1 surveys with a TF prevalence of <5.0%, 5.0 − 9.9% and ≥10.0%, respectively. All nine of the EUs with a TIS1 TF prevalence of 5.0 − 9.9% were resurveyed in the study period. Of those TIS2 results, the prevalence of TF was <5.0% in six TIS2 surveys and 5.0 − 9.9% in the remaining three (Figure 1). The change in TF over time is shown in (Figure 2).

A total of 67,407 participants aged ≥15 years were examined across all 48 surveys. In the 36 surveys conducted using version 2 of the Tropical Data protocol, we identified 774 cases of trichiasis (upper and/or lower eyelid) from 46,203 people examined. 576/774 (74%) of those cases were unknown to the health system and 524/774 (68%) had evidence of TS. The prevalence of TT (upper and/or lower eyelid) unknown to the health system was ≥0.2% in 24 of the 36 EUs surveyed using the version 2 protocol.

**Figure 3:**
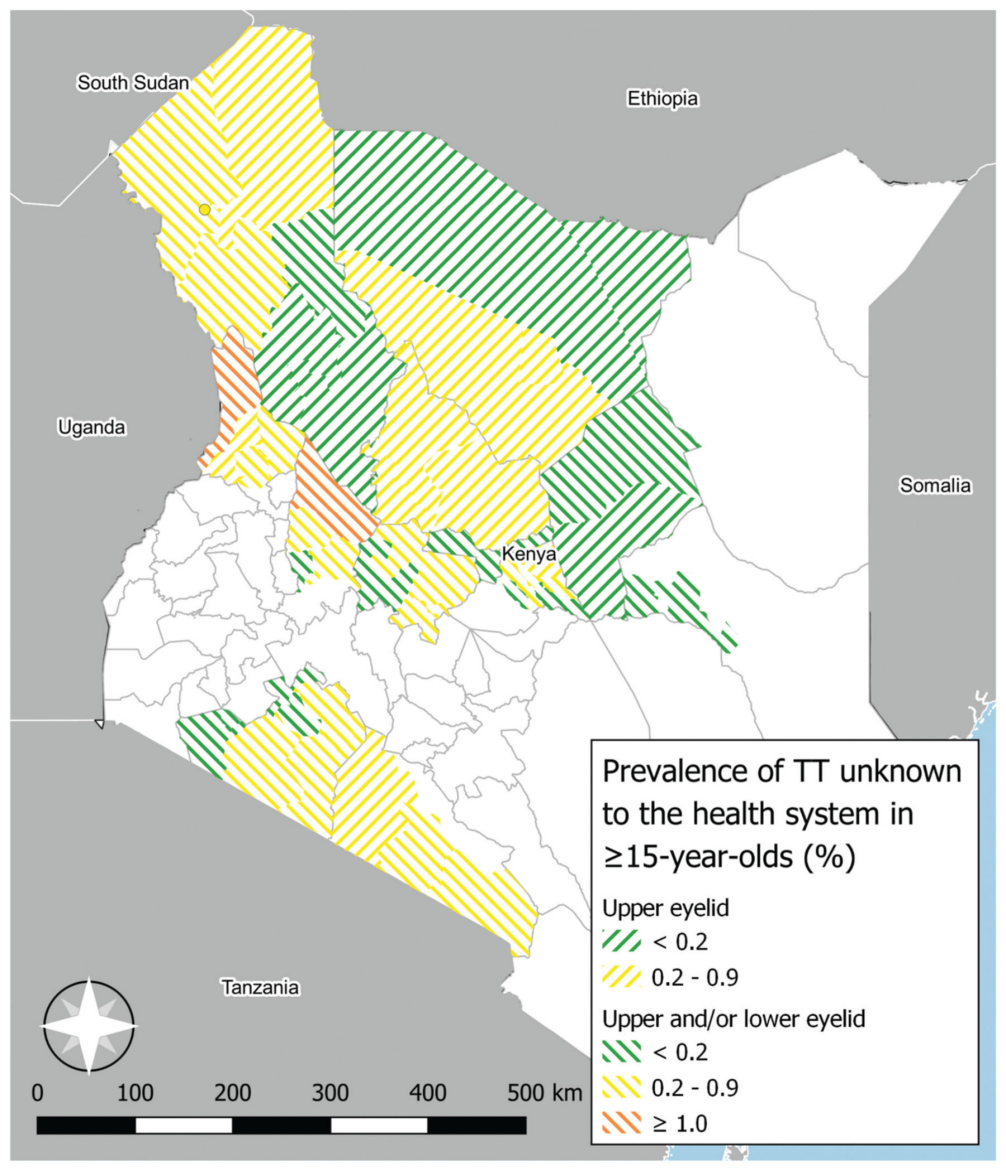
Adjusted prevalence of trachomatous trichiasis (TT) unknown to the health system in Kenya. Where more than one survey took place over the study period (April 2017–December 2020), only the most recent result is shown. Counties coloured in white were not surveyed. The boundaries and names indicated and the designations used on this map do not imply the expression of any opinion of any kind on the part of the authors, or the institutions with which they are affiliated, concerning the legal status of a country, territory, city or region or its authorities, or concerning the delimitation of its borders.

We conducted 12 surveys using version 3 of the Tropical Data protocol. In these 21,204 adults were examined, of whom 172 had upper-eyelid trichiasis. 127 (74%) upper-eyelid trichiasis cases were unknown to the health system and 121 (70%) had TS. The prevalence of TT (upper eyelid only) unknown to the health system was ≥0.2% in eight EUs (Figure 3, Supplementary Table S3).

**Figure 4:**
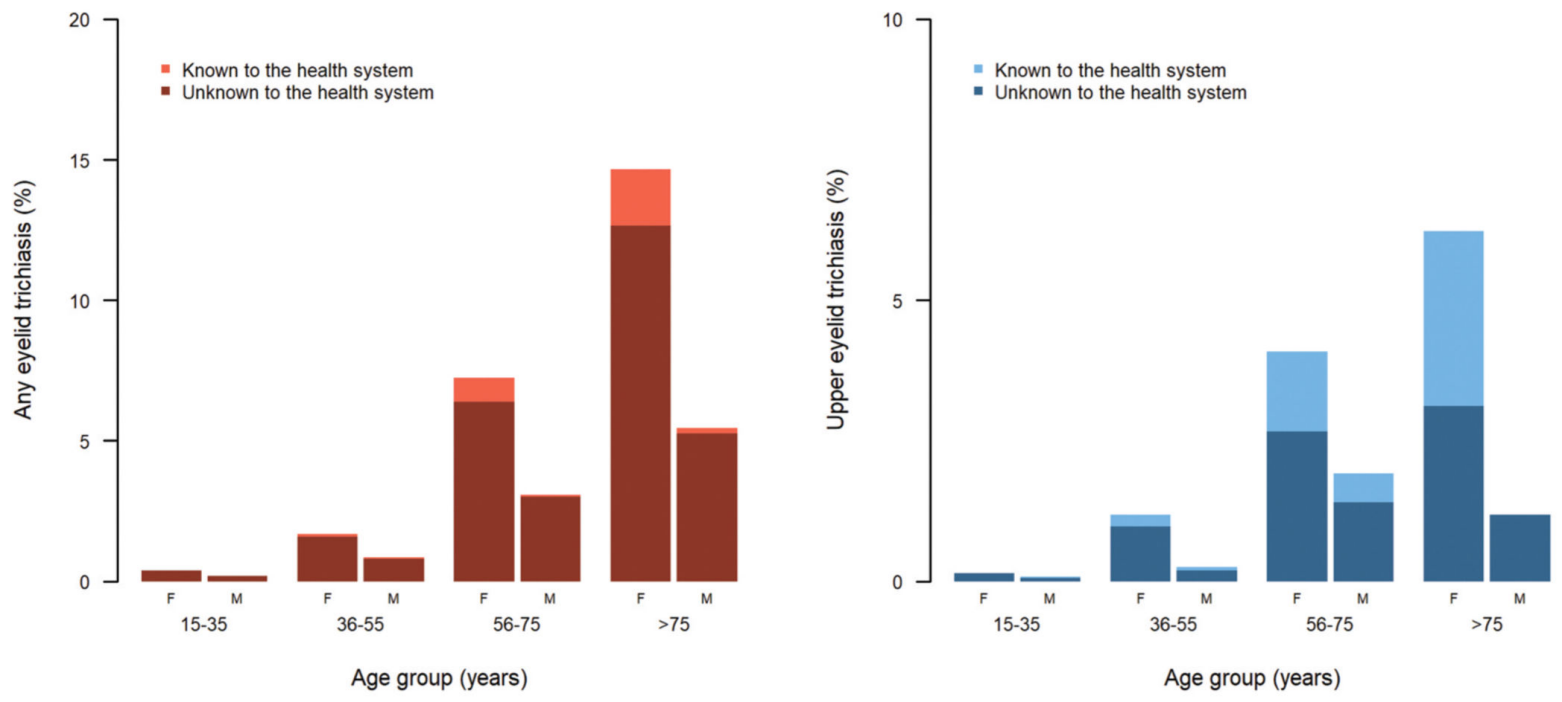
Age-specific trichiasis prevalence, separated by gender and whether the individual reported having been offered management in at least one affected eye. A. Data from 774 trichiasis cases identified during population-based prevalence surveys for trachoma in Kenya from 2017–2019. B. Data from 172 trichiasis cases identified during population-based prevalence surveys for trachoma in Kenya in 2020. F= female; *M* = male.

TT was much more common in older people than in younger people and was more common in females than in males (Figure 4). There was marginal evidence that more men with TT were ‘unknown to the health system’ than women with TT (*n* = 946 TT cases according to contemporary definition; *χ*^2^ = 3.63, *p* = .057).

### Household-level water and sanitation access

**Figure 5:**
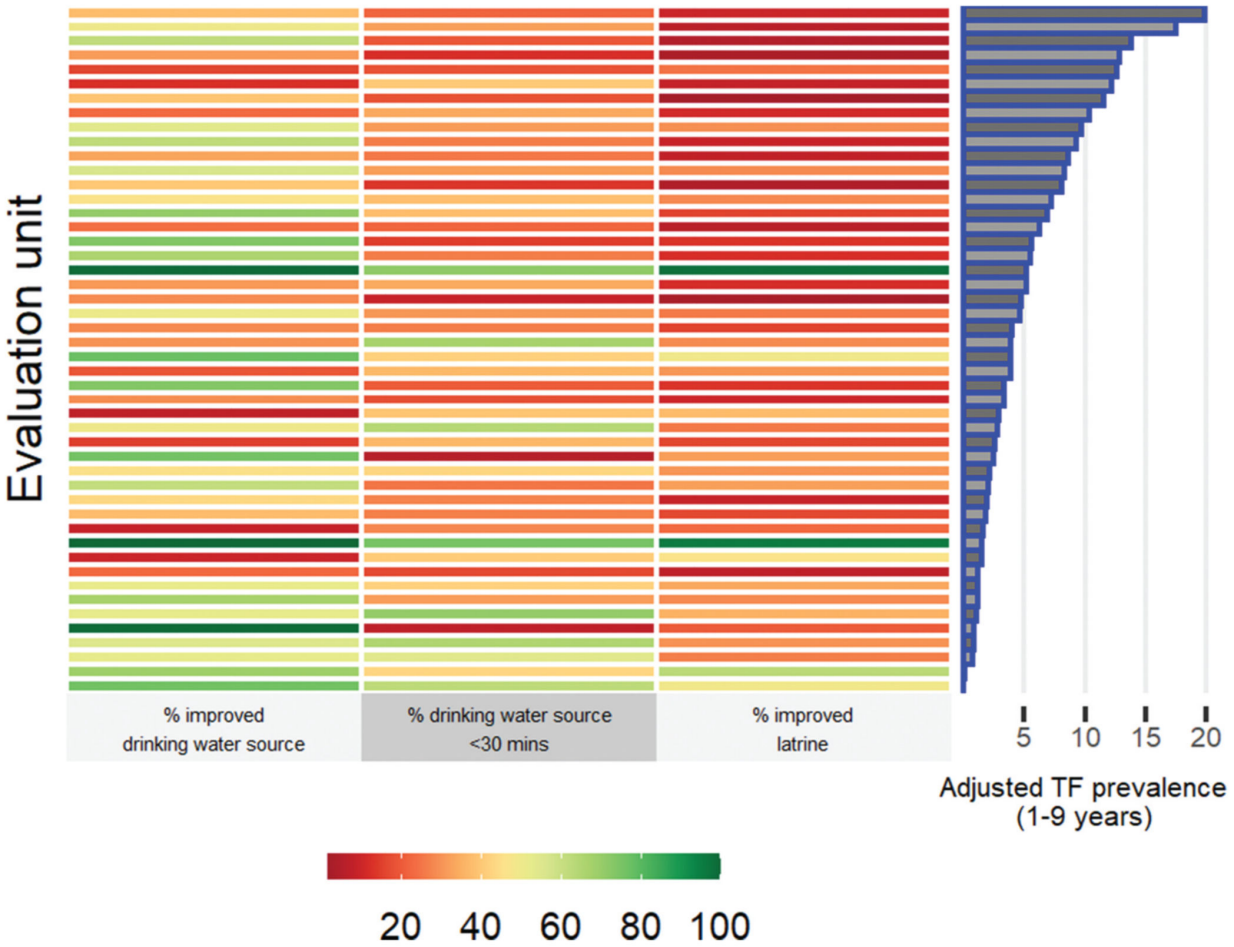
Proportion of households in each surveyed evaluation unit with access to improved water source and sanitation facilities, ordered by adjusted trachomatous inflammation—follicular (TF) prevalence in children aged 1–9 years. Data collected during trachoma surveys in Kenya, April 2017−December 2020.

Teams visited 42,789 households across the 48 surveys reported here (Figure 5, Supplementary Table S4). The proportion of households per survey with an improved drinking water source was highly variable: the median was 48% and the range 7 − 100%. The median proportion of households per survey with a drinking water source within a 30-minute round trip of the house was 31% (range: 6 − 75%). Sanitation and hygiene facilities were generally sub-optimal. The median proportion of households per survey with an improved latrine was 21% (range: 2–98%). In the version 2 protocol surveys, the median proportion of households per survey with a latrine with a handwash station within 15 m of the latrine was 1% (range: 0 − 33%). In the version 3 protocol surveys, the median proportion of households with a handwash station in or near the dwelling was 1% (range: 0 − 33%).

### Variables associated with trachomatous inflammation—follicular

There was strong evidence that TF was less common in older children (compared to 1−3-year-olds, the adjusted odds ratio [aOR] for TF in 4−6-year-olds and 7−9-year-olds was 0.64 [95% CI: 0.59 − 0.69; *p* < .001] and 0.10 [95% CI: 0.10 − 0.10; *p* < .001], respectively). There was strong evidence that TF was less common in households with a water source <30 minutes from the house (aOR: 0.77 [95% CI: 0.70 − 0.86], *p* < .001) but much more common in households practicing open defecation (aOR: 2.56 [95% CI: 2.21 − 2.96], *p* < .001).

## Discussion

East Africa has a significant proportion of the global burden of trachoma.^28^ Here, we present data from over 145,000 individuals surveyed by the Kenya National Trachoma Programme over a four-year period. The scale and integrity of this work is testament to the sustained commitment by the Kenya Ministry of Health and their partners to the elimination of trachoma as a public health problem.

The data highlight areas for optimism: in 12 baseline surveys, 10 NBLs and one TBL had a TF prevalence <5%. No further interventions or surveys are required there (though surgical programmes and TT case-finding should continue). Of 27 TIS1 surveys, 11 had a TF prevalence <5%. MDA should stop in these EUs and a surveillance survey should be conducted after two years. (In some cases, this has happened already.) There were nine TIS2 surveys of which six returned a TF prevalence of <5%. While MDA should stop in these areas, F and E activities should continue to ensure the low prevalence of trachoma is sustained.

The data presented also highlight areas where further work is needed. One NBL survey returned a TF prevalence ≥10%. Three rounds of MDA are needed in Narok Segment 2 before an impact survey is carried out. There were 16 TIS1 surveys which had a TF prevalence ≥5.0%. These EUs required between one and three rounds of MDA before resurveying; the data from nine of these are presented in this manuscript. There were three TIS2 surveys where the prevalence of TF was 5.0–9.9%. These are more concerning as the recommended number of rounds of MDA has been delivered twice and the prevalence of TF has still not fallen below the threshold for elimination. These EUs therefore qualify as having “persistent TF”, and could benefit from the recommendations of the *WHO Informal Consultation on End-Game Challenges for Trachoma Elimination*, including tailored management. This involves ensuring high-quality implementation of A, F and E with maximal coverage in all parts of an EU and the collection of data on conjunctival *C. trachomatis* infection and anti-*C. trachomatis* antibodies.^29^ F and E activities should continue in all EU regardless of the TF prevalence and/or MDA implementation.

The prevalence of TT unknown to the health system was ≥0.2% in 25 EUs. Increased provision of TT surgical services as well as continued case-finding is required in these areas, just as it is in Embu and Kitui counties, where recently published TT-only surveys also demonstrated above-threshold TT prevalence estimates.^30^ In Pokot North, the prevalence of TT unknown to the health system was >4%, which is extremely high. Given that many of the high TT prevalence EUs were surveyed before the definition of TT was changed to only include the upper eyelid, it is possible that lower eyelid trichiasis is partly responsible for the high overall prevalence. However, as West Pokot, which was surveyed using the new upper-eyelid only TT definition, also has relatively high TT prevalence (0.75%), we believe that this area does have a generally high TT prevalence, possibly due to fewer TT management opportunities. Pokot North should receive special attention in service planning. Interestingly, we found borderline evidence for males being less likely to have received TT management. It is important to ensure there is gender equity in provision of surgical services.^31^

Data on WASH access in surveyed areas were broadly comparable to previous estimates from Kenya. A recent United Nations JMP report on WASH infrastructure estimated that in 2015, 50% of rural house-holds in Kenya had access to an improved drinking water source within a 30-minute return journey of their household^32^; 22% of households in this series of surveys reported meeting that standard. The same JMP report estimated private, improved latrine access to be 27% among rural households in Kenya in 2015.^32^ In this survey series, 24% of households reported having access to an improved latrine. Accepting that a proportion of this variation may be due to methodological differences between direct data from surveys designed to measure trachoma and extrapolation methods used to estimate national-level WASH infrastructure coverage, the two data sources present a consistent picture. Unfortunately, that picture is one of very limited access to WASH infrastructure here. Inflammatory trachoma was more common in houses with the poorest WASH infrastructure, with odds of having TF two or three times higher in households in which open defecation was practiced relative to households with access to improved latrines, reinforcing the notion that this disease disproportionately affects under-served communities. It has been shown that open defecation provides breeding grounds for *Musca sorbens*, with female *Musca* spp. flies preferentially laying eggs on exposed human faeces.^33^ WASH improvements are urgently needed here, not just to reduce transmission of ocular *C. trachomatis* but to make progress towards the United Nations Sustainable Development Goals.

There were limitations to this study. The protocols used differed from the survey protocol used in previous survey rounds. One major difference is the definition of sampling frame from which clusters were selected. For example, in baseline surveys in 2004, the sampling frame was a county, whereas for the impact surveys of the same areas (presented here) there were multiple Eus per county. These and other methodological differences require caution to be exercised when directly comparing prevalence estimates over time. However, all protocols were recommended by WHO and were therefore appropriate at the time the surveys were conducted. From a forward-looking programme planning perspective, the data are valid.

A number of challenges remain for the Kenya National Trachoma Programme. Many are shared by other countries aiming to eliminate trachoma as a public health problem. The first is, as described above, how to improve F and E where WASH infrastructure coverage is limited. Strengthening the relationship between WASH and health partners will help to overcome this challenge.^34^ Second, 10/27 TIS returned TF prevalences ≥5% despite several rounds of MDA. The association between TF and ocular infection with *C. trachomatis* is known to deteriorate after MDA^35^ so further research into the prevalence of infection in these settings is warranted. Third, the majority of participants with trichiasis identified during this survey reported never having been offered management. Scale-up of surgical service provision, as well as continued detection of TT cases is needed in many trachoma-endemic counties. Fourth, many of the highest prevalence EUs border other countries; unregulated cross-border migration may be a source of re-introduction of trachoma to formerly endemic environments in both Kenya and its bordering countries, creating significant challenges for MDA implementation and trachoma surveys in all countries involved. Cross-border partnerships could play a key role in coordinating, aligning and intensifying regional trachoma elimination efforts.^36^ Indeed, in 2021, the first cross-border MDA for trachoma was undertaken in Kenya and Uganda, followed by cross-border MDA in collaboration with the United Republic of Tanzania in July 2022.^36^ Finally, detailed methodological recommendations for post-elimination surveillance in those EUs in which elimination targets have been met, whilst waiting for the country as a whole to receive validation of trachoma elimination as a public health problem, have not yet been released by WHO.

## Conclusion

These data provide a clear route forward for interventions in the short term and also highlight challenges that the programme must overcome before trachoma can be eliminated as a public health problem in Kenya. The prevalence of trachoma identified in these surveys suggests that Kenya still has some work to do, but this should in no way divert attention from the incredible progress that has been made, or the scale of the effort put into surveillance and interventions that has already been invested.

## Footnotes

Supplemental data for this article can be accessed online at https://doi.org/10.1080/09286586.2023.2280987
